# The effect of social anxiety on teenagers’ internet addiction: the mediating role of loneliness and coping styles

**DOI:** 10.1186/s12888-024-05854-5

**Published:** 2024-05-27

**Authors:** Wanglin Dong, Haishan Tang, Sijia Wu, Guangli Lu, Yanqing Shang, Chaoran Chen

**Affiliations:** 1 Institute of Nursing and Health, College of Nursing and Health, Henan University, Henan, Kaifeng, China; 2 Institute of Business Administration, School of Business, Henan University, Jinming Avenue, Henan, Kaifeng, 475004 China; 3 School of Basic Medical Science, Henan University, Jinming Avenue, Henan, Kaifeng, 475004 China

**Keywords:** Mental health, Addictive behavior, Anxiety, Coping styles

## Abstract

**Background & Aim:**

There is a lack of understanding of how social anxiety may affect Internet addiction among adolescents. Based on several theories, the purpose of this study was to investigate the multiple mediating roles of loneliness and coping styles in the association between social anxiety and Internet addiction in Chinese adolescents.

**Methods:**

This study used the Social Anxiety Scale, Internet Addiction Test, Loneliness Scale, and Simple Coping Style Questionnaire to investigate 1188 students in two junior high schools and senior high schools in Henan Province, China. We adopted Pearson’s correlation analysis and the PROCESS Macro Model 81 in regression analysis to explore the relationships among social anxiety, loneliness, coping styles, and Internet addiction.

**Results:**

We found that social anxiety not only directly affects teenagers' Internet addiction, but also affects teenagers' Internet addiction through loneliness and coping styles.

**Conclusions:**

These results emphasize the importance of improving social anxiety to reduce Internet addiction among adolescents. At the same time, it also emphasizes the need to reduce adolescents' loneliness and cultivating positive coping styles. In addition, this study has certain theoretical significance for teenagers' mental health and intervention studies on Internet addiction.

## Introduction

Internet addiction (IA) is not only a behavioral addiction, it is also considered an impulse control obstacle [1], which is mainly manifested in various online behaviors (online games, social networks, online shopping, watching pornographic websites, etc.) becoming uncontrolled and having a series of negative consequences for society, families, and individuals [2]. The latest version of the International Classification of Diseases (ICD-11) excludes IA in mental illness. However, it has included Internet gaming disorder as part of a “mental disorder caused by Internet addictive behavior” [3]. As adolescents access the Internet more than any other age group and undertake a higher risk of overuse of the Internet, the problem of IA is most relevant to young people [4]. Recent survey for IA showed that the IA rate of teenagers is as high as 10% in China, and the prevalence of IA among adolescents in 11 European countries was found to be 4.4% [5]. It indicates prevalence varied significantly across different countries. The research on teenagers and IA shows that IA can lead to bad habits (eating disorders, work, and rest disorders, smoking, etc.), decreased academic performance (reduced attention and memory), and anxiety and depression in some mental diseases [6, 7]. And with time, non-suicidal self-injury and suicidal ideation of teenagers with Internet addiction showed an upward trend [8, 9]. Therefore, teenagers’ IA is a concern in China and many other countries. It is important to note that social anxiety is a predictor of internet addiction in adolescents. According to the self-regulation hypothesis and compensation hypothesis according to which socially anxious individuals may use the internet to cope up with the fear of social interactions as it offers a virtual platform for interaction. Therefore, we need to understand the mechanisms of social anxiety on Internet addiction and formulate effective preventive intervention measures to reduce the occurrence of IA in teenagers. Based on previous study, it is found that more studies attention to the influencing factors of social anxiety and internet addiction among teenagers. In this study, loneliness and coping styles are added, hoping to reveal the intermediary mechanism between social anxiety and internet addiction through the theoretical model between social anxiety, loneliness, coping style and internet addiction. So as to enrich and improve the related research on adolescent Internet addiction, and provide reference for formulating intervention programs for adolescent Internet addiction.

## Background

### Effect of social anxiety on *IA*

Social anxiety is a type of general anxiety that refers to negative emotional experiences and behavioral manifestations such as fear, embarrassment, and avoidance that an individual produces in imagined or real social situations [10]. Social anxiety is considered as an influencing factor of Internet addiction [11–13]. The general model of addiction shows that a person's choice of substances is closely related to the specific painful emotional state they are trying to control [14]. Teenagers with social anxiety face greater psychological stress in their life, they often prefer to spend time engaging in activities alone, including surfing the internet and prefer to interact with others online as opposed to in person [15]. The anonymity and convenience of the Internet provide non-face-to-face way communication for adolescents with social anxiety to neutralize or avoid threats in real-world social situations through online communication [16]. However, individuals who are addicted to online social interaction may have reduced social skills, fear of real social interaction, and aggravate social anxiety due to ignoring real-life situations and lack of face-to-face communication opportunities [17]. In this study, we propose hypothesis 1, that social anxiety positively related to adolescent IA behavior.

### The potential mediating role of loneliness

Loneliness is an important public health problem in society, with surveys showing that 6–8% people are affected by loneliness [18]. Loneliness is a negative affective state caused by the difference between the individual's desired level with the actual level of interpersonal relationships, mainly including social loneliness and emotional loneliness [19]. The occurrence of loneliness is associated with many factors such as low mood, hopelessness, depression, anxiety, and low sense of self, among others [20]. Among them, the relationship between anxiety and loneliness has long been studied. Studies have shown that both general anxiety and social anxiety are associated with loneliness and that social anxiety is the another important predictor of loneliness besides depression and general anxiety [21]. The cognitive and behavioral model of social anxiety explains the process of loneliness caused by social anxiety, and social anxiety will make negative assessments of social situations, doubt their social skills, and will experience nervousness and anxiety in social situations, so as to choose social avoidance behaviors. Prolonged social avoidance reduces the communication and interaction between socially anxiety individuals and others, thereby increasing the social loneliness and emotional loneliness of individuals. However, both social anxiety and loneliness can increase frustration and reduce social belonging in adolescents. According to the decompensation hypothesis, when adolescents are hindered in their development, they may choose to use the Internet to relieve stress and compensate. Many scholars have conducted research on the relationship between loneliness and addictive behavior. Mehmet Emin Parlak investigated 634 middle school students, and the results showed that there was a significant positive correlation between Internet addiction and adolescent loneliness [22]. According to the results of a survey of 582 Chinese college students by Yanhong Zhang et al., Loneliness was significantly and positively associated with mobile phone addiction [23]. In addition, studies have shown that lonely and depressed individuals prefer online social interaction over face-to-face communication, leading to increased Internet use or compulsive Internet use in adolescents, increasing the incidence of IA in adolescents [24]. Therefore, this study proposes hypothesis 2 that loneliness mediates between social anxiety and IA.

### The potential mediating role of coping styles

Coping styles refer to an individual's cognitive and behavioral efforts to alleviate stress [25]. According to different coping styles, there are positive coping styles, coping strategies such as actively seeking help, problem-solving, and reconstructing. And negative coping styles, coping strategies such as self-blame, avoidance, and fantasy. The quality-stress model theory suggests that when two people face the same pressure, more vulnerable people are more likely to have negative attitudes. Dong Z et al. showed that problem-solving styles showed a small negative link with social anxiety (*r* = -0.198) [26]. Yang T survey of 2695 college students showed that positive coping styles was negatively associated with social anxiety [27]. Therefore, adolescents with social anxiety are psychologically threatened in social situations and usually adopt a negative and avoidant coping style. When adolescents develop negative avoidance coping attitudes or behaviors, will be more likely to lead to overuse of the Internet. Shan X et al. survey of 3,380 first-year college students in South China showed that the addiction group adopted less positive coping style (*p* < 0.05) and preferred negative coping style (*p* < 0.05) than non-addiction group [28]. Yi X et al. survey of 1545 middle-school students showed that positive coping styles had a significant negative predictive effect on the random intercept of IA, while negative coping style had a significant positive predictive effect on the random intercept of IA [29]. Therefore, coping styles may be significantly associated with adolescent IA. Based on previous theories and studies, we propose hypotheses 3, the mediating role of coping styles between social anxiety and IA (H3).

Cognitive interaction theory states that factors such as the environment, stressors, and subjective cognition all affect coping styles, with individual cognitive factors playing the strongest role. Adolescents with higher loneliness experiences will be accompanied by negative emotional states such as low self-esteem and depression, and their cognition of things is more inclined to be negative [30, 31]. Some studies demonstrated that with loneliness positively correlated with negative coping styles and negatively correlated with positive coping styles [32, 33]. Therefore, we propose hypotheses 4, the mediating role of coping styles between loneliness and IA (H4).

### Study framework

**Fig.1:**
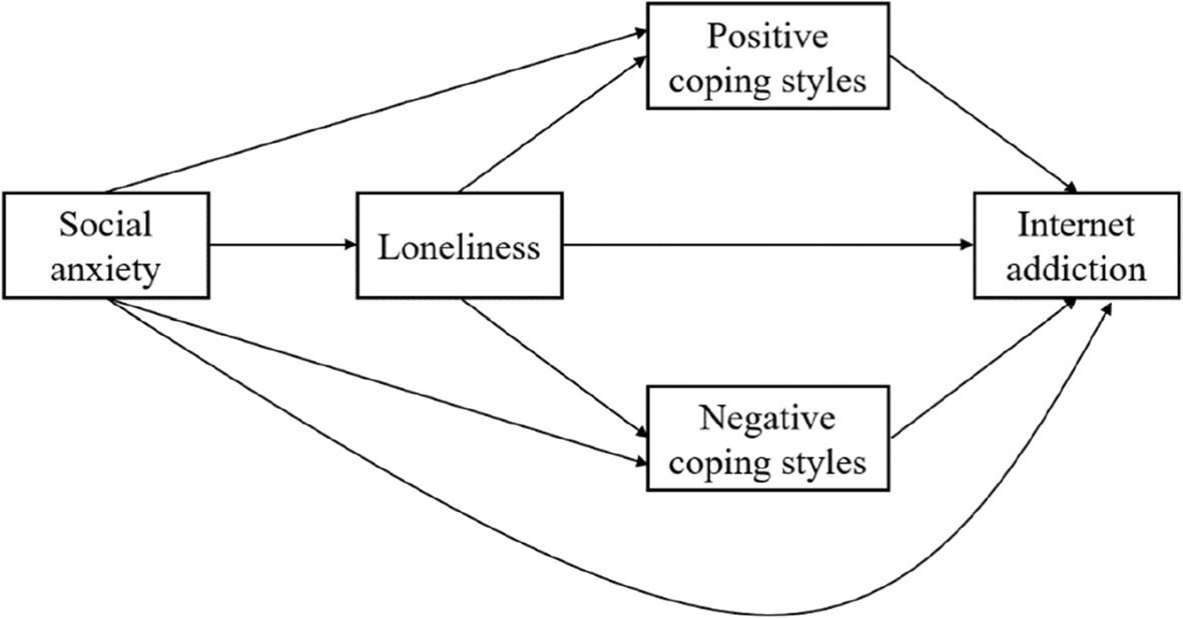
Conceptual framework

Social anxiety, loneliness, coping styles, and IA interact with each other. Nevertheless, it is not clear how these variables interact with each other to cause IA in teenagers. In this study, we put forward the conceptual framework as shown in Fig. 1. This study discusses the influence of social anxiety on IA from the perspective of teenagers, and discusses the role of loneliness and coping style, in order to provide some theoretical support and guidance for the related research and intervention of teenagers' psychological and behavioral health.

## Method

### Demographic data

From May to June 2023, volunteers were recruited from two junior and senior high schools in Henan Province, China, using a convenient sampling method. Participants accord with the following inclusion criteria: (1) enrolled secondary school students; (2) informed consent and voluntary participation in this study. The exclusion criteria: (1) not in school during the investigation; (2) answering the questionnaire regularly; (3) due to various reasons, all the contents of the questionnaire were not completed. The study distributed a total of 1300 questionnaires and recovered 1208 questionnaires, with a recovery proportion of 92.92%. Excluding 20 questionnaires with incomplete answers, we recovered 1188 valid questionnaires, with a valid recovery proportion of 91.38%. We investigated volunteers' gender, grade, parents' marriage, family location, parents' education level, and relationships with teachers and students.

### Social anxiety scale

The social anxiety scale was used the Social Anxiety Inventory (SAS-A) which compiled by La G [34], Chinese scholar Zhu H translated and revised the Chinese version in 2008 [35]. The scale consists of 13 items, including the following three dimensions: fear of negative evaluation (6 items), social avoidance and distress in an unfamiliar situation (4 items) and social avoidance and distress in general situations (3 items). It adopts the Likert five-point scoring method, which ranges from 1 (completely inconsistent) to 5 (fully compliant). The total score of the scale entries can be understood as the initial indication of the degree of social anxiety of the participant. The higher the score on this scale, the higher the social anxiety level of the individual. Cronbach’s a for this scale in this study was 0.899.

### Loneliness scale

The loneliness scale was used University of California Los Angeles Loneliness Scale (ULS) which compiled by Russell in 1978 [36]. It has been validated in China [37]. The scale consists of 20 items, including 11 positive scoring questions, and 9 reverse scoring questions. It adopts the Linkert four-point scoring method, which ranges from 1 (never) to 4 (always). The higher the score, the stronger the individual's experience of loneliness. The Cronbach's α coefficient on this study scale was 0.848.

### Coping styles scale

The coping styles scale used a Simple Coping Style Questionnaire (SCSQ) which compiled by Xie Y based on the characteristics of the Chinese [38]. The scale contains two types of coping styles: positive coping styles (12 items) and negative coping styles (8 items). Each item uses a Likert four-point scoring method, which ranges from 1 (do not use) to 4 (often use). The higher the score of the positive coping dimension, the more positive the individual’s coping styles; the higher the score of the negative coping dimension, the more negative the individual’s coping styles. The scale in this study had a Cronbach's α coefficient of 0.789. Cronbach's α for positive coping styles was 0.823 and Cronbach's α for negative coping styles was 0.729.

### Internet addiction scale

The IA scale used Internet Addiction Test (IAT) which compiled by Kimberly Young [39]. The scale is a self-rating scale, containing 20 items. It has been validated in various countries including China [40]. Each item uses a Likert five-point scoring method, which ranges from 1 (never) to 5 (always), the total score range is 20–100 points. The score ≥ 50 points can be judged as IA, the higher the score, the more serious the problem of IA. The Cronbach's α coefficient on this study scale was 0.902.

### Ethical statement

This study recruited volunteers with informed consent and voluntary participation for anonymous surveys. In addition, this study was supported by the ethics of relevant institutions (20230516001).

### Statistical techniques

IBM SPSS Statistics 26.0 and the PROCESS Macro (v4.1 by Andrew F. Hayes) was used for statistical analysis in this study. First, the participants’ demographic characteristics and the IA, social anxiety, loneliness, and coping styles scores were measured using descriptive statistics (percentages, means, standard deviations, etc.). Secondly, kurtosis and skewness were used to test the normality of social anxiety, Internet addiction, loneliness, and coping styles. If social anxiety, loneliness, coping styles, and IA follow a normal distribution, we use Pearson's correlation analysis to explore the relationship between these variables. Otherwise, we used Spelman for the analysis. Then we used PROCESS Model 81 (v4.1) to examine the mediating role of loneliness and coping style between social anxiety and Internet addiction. Finally, we calculated 95% confidence intervals for bias-corrected percentile bootstrapping through a bootstrapped sample of 5000. The P-value is two-tailed, below 0.05, and statistically significant.

## Results

### The demographic characteristics of the participants

All the 1,188 volunteers are from junior high schools or high schools, including 696 males (58.6%) and 492 females (41.4%). There are 224 parents with disharmony in marriage, accounting for 18.9%. 675 students are living in rural areas and 513 students living in towns. The educational level of fathers (58.7) and mothers (66.7) is mostly in junior high school and below. In school life, 2.8% have a bad relationship with classmates and 4.4% have a bad relationship with teachers. See Table 1 for the detailed description and statistical results.

**Table 1:** Demographic characteristics of the participants (*n* = 1188)

Variable	Option	*n*	%
Gender	Male	696	58.6
	Female	492	41.4
Grade	Junior students	577	48.6
	Senior students	611	51.4
Parents' marriage	harmonious	964	81.1
	disharmony	224	18.9
Residence	Village	675	56.8
	Town	513	43.1
Father's education level	Junior high school and below	697	58.7
	high school and above	494	41.3
Mother's education level	Junior high school and below	792	66.7
	high school and above	396	33.3
Relationship with classmates	Poor	33	2.8
	General	579	48.7
	Good	576	48.5
Relationship with teachers	Poor	52	4.4
	General	761	64.1
	Good	375	31.6

### Assessment of common method bias

Because all data were collected with questionnaires, we used Harman's single-factor test for possible common method bias. The test results showed that there were 14 variables with eigenvalues greater than 1. The first variable explained 17.41% of the total variation, which is below the critical standard of 40% [41]. Hence, there were no serious common methodological biases in this study*.*

### Pearson’s correlation analysis

The correlation coefficients, mean ± standard deviation, skewness, and kurtosis results of each variable in this study are shown in Table 2. The scale scores of adolescents for IA, social anxiety, positive coping styles, negative coping styles, and loneliness in this study were 2.381 ± 0.66, 2.618 ± 0.85, 2.571 ± 0.54, 2.167 ± 0.58 and 2.273 ± 0.47, respectively.

**Table 2:** Descriptive statistics and correlations of the study variables (*n* = 1188)

Variables	1	2	3	4	5	M ± SD	*S*	*K*
1. IA	1					2.381 ± 0.66	.581	.359
2. SA	.431**	1				2.618 ± 0.85	.240	-.343
3. PCS	-.211**	-.225**	1			2.571 ± 0.54	.102	.176
4. NCS	.308**	.241**	.145**	1		2.167 ± 0.58	.335	-.185
5. Loneliness	.347**	.432**	-.358**	.197**	1	2.273 ± 0.47	.106	.135

*IA* Internet Addiction, *SA* Social Anxiety, *PCS* Positive Coping Styles, *NCS* Negative Coping Styles, *S* Skewness, *K* Kurtosis

^**^p < 0.01

In this study, we used *Pearson* correlation analysis to perform an exploratory analysis of the correlation between individual variables. Correlation analysis found that there is a significant correlation between the variables in this analysis, and all of them are significant at the significance level of 99%. There was a positive relation between IA and social anxiety (*r* = 0.431, *p* < 0.01), and negative coping styles (*r* = 0.308, *p* < 0.01), and loneliness (*r* = 0.347, *p* < 0.01); IA and positive coping styles were significantly negatively correlated (*r* = -0.211, *p* < 0.01). In addition, there was an association between social anxiety and positive coping styles (*r* = -0.225, *p* < 0.01), negative coping styles (*r* = 0.241, *p* < 0.01), and loneliness (*r* = 0.432, *p* < 0.01). Moreover, we also found a relation between loneliness and positive coping styles (*r* = -0.358, *p* < 0.01), and negative coping styles (*r* = 0.197, *p* < 0.01).

### Mediating effect analysis

The multiple linear regression analysis discovered that grade, the mother's education level, the marriage situation of the parents, and the relationship with teachers had a significant influence on the IA of students. Therefore, these were used as the control variable in mediating effect analysis.

**Fig. 2:**
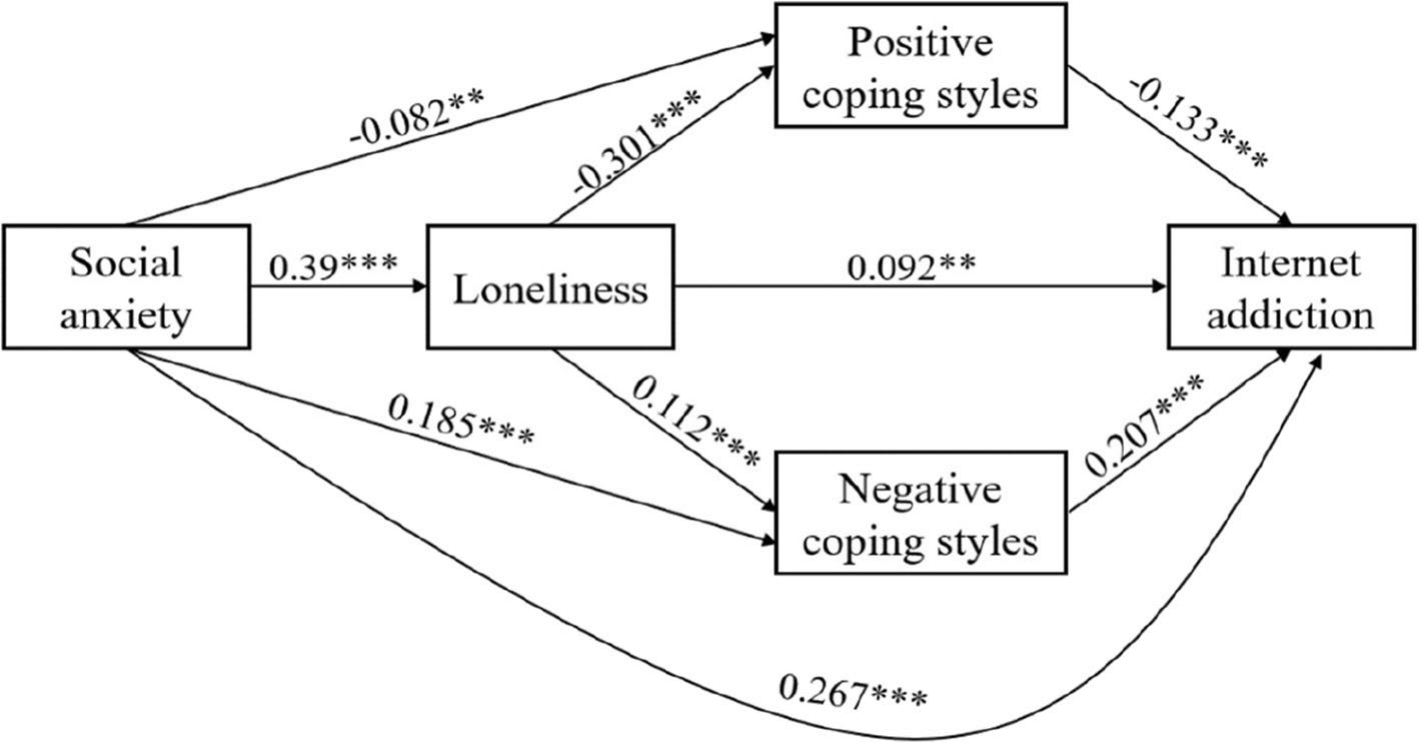
Social anxiety and Loneliness and coping styles of mediating role affect Internet addiction. **p < 0.01, ***p < 0.001 (two-tailed)

We used the PROCESS Macro Model 81 (v4.1) for testing mediation hypotheses for regression analysis, the results are shown in Table 3 and Fig. 2. Social anxiety significantly positively related to loneliness (*β* = 0.39, *p* < 0.001), negative coping styles (*β* = 0.185, *p* < 0.001), and IA (*β*= 0.267, *p* < 0.01). But social anxiety significant negative related to positive coping styles (*β*= -0.082, *p* < 0.01). Loneliness significantly positively related to negative coping styles (*β*= 0.112, *p* < 0.001) and IA (*β*= 0.092, *p* < 0.01). And loneliness significant negative related to positive coping styles (*β*= -0.301, *p* < 0.001). Positive coping styles significantly negatively related to IA (*β* = -0.133, *p* < 0.001). Negative coping styles significantly positively related to IA (*β* = 0.207,* p* < 0.001).

**Table 3:** Mediation effect test for the regression analysis (*n* = 1188)

Model	M1(dependent variable: Loneliness)		M2(dependent variable: PCS)		M3(dependent variable: NCS)		M4(dependent variable: IA)	
Predictive variable	*β*	*t*	*β*	*t*	*β*	*t*	*β*	*t*
Constant	2.010	23.610***	3.161	25.802***	1.248	8.980***	1.278	7.486***
Grade	0.073	2.8338**	0.102	3.786***	0.138	4.891***	0.227	9.244***
Mother's education level	-0.024	-0.939	0.101	3.788***	0.060	2.147*	-0.053	-2.17*
Relationship with teachers	-0.198	-7.599***	0.082	2.972**	0.014	0.499	-0.077	-3.098**
Marriage situation of the parents	0.035	1.353	-0.071	-2.655**	-0.009	-0.323	0.087	3.601***
SA	0.390	14.960***	-0.082	-2.752**	0.185	5.989***	0.267	9.824***
Loneliness			-0.301	-9.935***	0.112	3.537***	0.092	3.221**
PCS							-0.133	-4.957***
NCS							0.207	8.034***
R-sq	0.23		0.168		0.091		0.331	
*F*	70.627***		39.600***		19.680***		72.776***	

*IA* Internet addiction, *SA* Social anxiety, *PCS* Positive coping styles, *NCS* Negative coping styles

^*^p < 0.05, **p < 0.01, ***p < 0.001 (two-tailed)

The mediating role of loneliness and coping styles in social anxiety and IA models was examined by Bootstrap test, we calculated 95% confidence intervals. According to the analysis results in Table 4, it can be found that the mediating effect consists of indirect effects generated by five pathways. Firstly, the pathway coefficient of social anxiety affecting IA through loneliness was 0.036 (0.012, 0.060). The pathway coefficient of social anxiety affecting IA through positive coping style was 0.011 (0.002, 0.021), the pathway coefficient of social anxiety affecting IA through negative coping style was 0.038 (0.022, 0.057), and the pathway coefficient of social anxiety affecting IA through loneliness and positive coping styles chain mediation was 0.016 (0.009, 0.024), the path coefficient of social anxiety affecting IA through the chain mediation of loneliness and negative coping styles was 0.009(0.004, 0.016). The total value of the indirect effect is 0.110 (0.081, 0.140), and the confidence interval does not contain 0, so the mediating indirect effect holds. So, loneliness and coping styles played a significant mediating effect in the model. The confidence interval for the direct effects test results also does not contain 0, indicating that the direct effects also hold, so loneliness and coping styles are partially mediating in the model. According to the calculation of the proportion of effects, it can be seen that the indirect effect of loneliness and coping style accounted for 29.18%.

**Table 4:** Bootstrap Mediation effect test (*n* = 1188)

Effects	Paths	Effect	SE	Bootstrap 95% CI
Total effect	SA → IA	0.377	0.020	0.256, 0.333
Direct effect	SA → IA	0.267	0.021	0.167, 0.250
Ind1	SA → Loneliness → IA	0.036	0.012	0.012, 0.060
Ind2	SA → PCS → IA	0.011	0.005	0.002, 0.021
Ind3	SA → NCS → IA	0.038	0.009	0.022, 0.057
Ind4	SA → Loneliness → PCS → IA	0.016	0.004	0.009, 0.024
Ind5	SA → Loneliness → NCS → IA	0.009	0.003	0.004, 0.016
Total indirect effect	\	0.110	0.015	0.081, 0.140

## Discussion

This study investigated 1188 teenagers in China, and analyzed the relationship between social anxiety, loneliness, coping styles and IA. These results verified the hypothesis that social anxiety has a positive related to IA, and can indirectly influence IA through loneliness and coping styles. To the best of our knowledge, this study discusses the relationship between social anxiety, loneliness, coping styles and IA of teenagers for the first time. In addition, the results also showed that compared with loneliness and positive coping styles, the mediating effect value of negative coping styles is the greatest in social anxiety and IA, which provided an empirical basis for the formulation of adolescent IA intervention programs in the future.

### Direct effects of social anxiety on adolescent *IA*

This study found that adolescents had a high average score on social anxiety scale (2.62 ± 0.85), which is consistent with previous studies [42, 43]. The results of this study also demonstrate a direct relationship between adolescent social anxiety and IA, and the H1 was validated. Sahar Obeid et al. investigated 1103 young adolescents aged between 13 and 17 years, and the results showed that social anxiety was associated with higher IA (β = 0.084) [44]. Bengü Yücens investigated 392 undergraduate medical students, and the results showed that IA group had significantly higher scores on social anxiety than the control group, and social anxiety was the strongest predictor of the severity of IA [14]. These studies are consistent with the results of this study, and general model of addiction was validated. Individuals with social anxiety have high interpersonal sensitivity and are very sensitive to external criticism and rejection [45]. Therefore, individuals with social anxiety are in a weak position to establish good relationships with others in reality, and will have a tendency to socialize in escapist situations. Use the Internet to communicate online that socially anxious individuals can avoid facing face-to-face social situations, reduce unnecessary tension, fear and embarrassment, and more easily obtain interpersonal support. The internet is a way for teens to alleviate social anxiety, but if self-regulation of online use is inadequate, it can be difficult to control the timing and frequency of use. When adolescents rely too much on online regulation, it can cause IA problems [46]. This reminds us that we must not only focus on the life and learning needs of adolescents, but also on the mental health. Schools can identify students with social anxiety through screening, and actively provide psychological counseling and social skills guidance. Help teenagers form a positive and healthy social psychology and avoid the occurrence of IA.

### Loneliness related mediation model

This study explores the association between adolescent social anxiety and IA, suggesting that loneliness plays a mediating role in the impact of social anxiety on IA, the hypothesis 2 was validated. The results showed that social anxiety had a significant related to loneliness on adolescent, which is consistent with previous studies [47, 48]. Loneliness is a common experience in adolescence. In this study, adolescents had a loneliness score of (2.27 ± 0.47), which was at a moderate level. According to one survey report, 11–20% of people aged 12–15 years feel lonely at least “sometimes” [49]. Adolescents with social anxiety lack self-confidence and security when it comes to socializing, limiting their ability to build harmonious interpersonal relationships with their peers, thereby exacerbating their loneliness [50]. The results also validated the cognitive and behavioral model of social anxiety, that is, adolescents with social anxiety choose avoidant socialization, which will increase psychological problems such as loneliness. A Study has shown that in addition to depression and computer self-efficacy, loneliness is also an important predictor of IA [51]. This study shows a positive correlation between loneliness and internet addiction, consistent with previous findings. Adolescents with higher loneliness have a lower sense of social belonging and self-identity. The Internet can provide adolescents with emotional value and security, so they tend to use online socializing to meet their sense of belonging [52]. The results of this study are consistent with the decompensation hypothesis of IA. However, excessive use of the Internet can cause IA among adolescents, affecting the life and academic performance of adolescents [53, 54]. Therefore, we should pay attention to finding students with social anxiety, timely propose the experience of correcting interpersonal relationships, help students actively integrate, and prevent or reduce the occurrence of adolescent loneliness.

### Coping styles related mediation model

This study shows that coping styles are related to factors such as grade, mother's education level, parents' marital status, and relationship with teachers. Coping style is influenced by a variety of factors. Families are an important source of support for adolescents in China [55]. The family environment (including parenting style, level of education, marital status) is closely related to the adolescent's coping style [5]. In addition, social support is also closely related to adolescents' ability to cope with stress [55, 56]. The teacher-student relationship is closely related to adolescent psychological symptoms (anxiety and depression) [57]. Therefore, when the teacher-student relationship is good, adolescents can feel higher social support and are prone to positive coping styles, while vice versa are prone to negative coping styles. Meanwhile, the results showed that coping styles played a mediating role in the influence of social anxiety on IA in adolescents, and hypothesis 3 was validated. Social anxiety positively predicts negative coping styles and is inversely correlated with positive coping styles. This suggests that social anxiety teenagers tend to use negative coping styles to solve problems, consistent with previous studies [58, 59]. Studies have shown that adolescents with social anxiety may experience interpersonal difficulties and form negative perceptions of themselves and others, leading to an increase in depression or aggressive behavior [60]. These adverse outcomes increase adolescents' negative perceptions, leading them to negative coping styles such as avoidance and self-harm. These results also validate the quality-stress model theory. In addition, the results showed an inverse correlation between positive coping styles and IA, and negative coping styles positively predicted IA, which is consistent with previous studies [28, 61, 62]. Therefore, it is important to actively guide adolescents to develop positive coping styles. For example, in terms of family, in addition to a harmonious and optimistic family atmosphere, parents should provide positive parenting methods, promote the formation of healthy psychological qualities in adolescents, and reduce the occurrence of social anxiety and IA.

This study also found that coping style is the mediating variable between adolescent loneliness and IA, and tested hypothesis4. The results show that loneliness has a positively related with negative coping style and is negatively correlated with a positive coping style. Previous studies suggest that lonely individuals may have more negative psychological states and less confident relationships, which can lead adolescents to adopt negative coping styles, consistent with the results of this study [33, 63]. Lonely adolescents are more inclined to find psychological comfort and safe interpersonal relationships on the Internet, which increases the occurrence of IA [64, 65]. Remind us that it is especially important to pay attention to the mental health of adolescents. The school's education system and basic health care centers should play a key role in spreading a positive attitude, timely intervention in adolescents' bad psychology and negative emotions, and reducing the occurrence of adolescents' loneliness and other bad psychology.

### The chain-mediating effect

In addition, the results of this study proved that adolescent social anxiety can affect IA through the chain mediating effect of loneliness and coping styles. Studies have shown that higher levels of social anxiety, greater loneliness, and a greater tendency to use negative coping styles such as avoidance, self-blame, and abandonment to deal with problems, thereby increasing the chances of IA [66–68]. However, when adolescents have a lower level of social anxiety, they get along better with each other, reduce the occurrence of loneliness, are more inclined to adopt positive problem-solving methods, and have a higher sense of subjective well-being to reduce the occurrence of addictive behaviors. This study provides a certain degree of theoretical support and guidance for the research and intervention of adolescent Internet addiction and is of great significance for promoting the healthy development of adolescent psychology and behavior.

## Limitations

First, this study only investigated two schools in one province, which somewhat hindered the generalizability of the conclusions. A national multi-center sampling survey should be conducted. Secondly, this study used the questionnaire method, and the subjective report may have some problems such as recall bias. Although no common methodological biases were found in this study, more objective data collection should also be considered in subsequent studies. Finally, this study is a cross-sectional survey and cannot reveal the causal relationship between variables, so a corresponding longitudinal study should be carried out later.

## Conclusion

This study constructs a chain intermediary model from the perspective of teenagers to explore the process and mechanisms of social anxiety influencing IA. The study discovered that social anxiety has a positive predictive effect on teenagers' IA, loneliness, and coping styles play a chain intermediary role in the influence of childhood on IA among teenagers. It verified the mediating model of social anxiety→loneliness→coping styles→IA of teenagers. Therefore, in order to improve teenagers' social anxiety, loneliness and IA, parents and schools are advised to pay more attention to teenagers' mental health and encourage teenagers to take positive coping styles to avoid social anxiety and IA.

## Data Availability

The datasets can be made available to any interested person(s) contacting the corresponding author via email.
